# A feasibility study comparing UK older adult mental health inpatient wards which use protected engagement time with other wards which do not: study protocol

**DOI:** 10.1186/s40814-016-0049-z

**Published:** 2016-01-29

**Authors:** Fiona M. Nolan, Chris Fox, Richard Cheston, David Turner, Allan Clark, Emily Dodd, Mary-Ellen Khoo, Richard Gray

**Affiliations:** 1 Centre for Outcomes Research and Effectiveness (CORE), University College London, 1-19 Torrington Place, London, WC1E 7HB UK; 2 Department of Clinical Psychology, Norwich Medical School, Faculty of Medicine and Health, University of East Anglia, Earlham Road, Norwich, NR4 7TJ UK; 3 Health and Social Sciences, University of the West of England, Glenside Campus, Bristol, BS16 1DD UK; 4 Department of Population Health and Primary Care, Norwich Medical School, Faculty of Medicine and Health, University of East Anglia, Earlham Road, Norwich, NR4 7TJ UK; 5 University of the West of England, Room 2G05, Glenside Campus, Blackberry Hill, Bristol, BS16 1DD UK; 6 Camden and Islington NHS Foundation Trust, 1st Floor East Wing, St Pancras Hospital, 4 St Pancras Way, London, NW1 0PE UK; 7 Hamad Medical Corporation, PO Box 3050, Doha, Qatar

**Keywords:** Mental health inpatient wards, Protected engagement time, Therapeutic engagement, Ward activity, Older adult

## Abstract

**Background:**

Protected engagement time (PET) is a concept of managing staff time on mental health inpatient wards with the aim of increasing staff and patient interaction. Despite apparent widespread use of PET, there remains a dearth of evidence as to how it is implemented and whether it carries benefits for staff or patients. This protocol describes a study which is being carried out on mental health wards caring for older adults (aged over 65) in England. The study shares a large proportion of the procedures, measures and study team membership of a recently completed investigation of the impact of PET in adult acute mental health wards. The study aims to identify prevalence and components of PET to construct a model for the intervention, in addition to testing the feasibility of the measures and procedures in preparation for a randomised trial.

**Methods/design:**

The study comprises four modules and uses a mixed methods approach. Module 1 involves mapping all inpatient wards in England which provide care for older adults, including those with dementia, ascertaining how many of these provide PET and in what way. Module 2 uses a prospective cohort method to compare five older adult mental health wards that use PET with five that do not across three National Health Service (NHS) Foundation Trust sites. The comparison comprises questionnaires, observation tools and routinely collected clinical service data and combines validated measures with questions developed specifically for the study. Module 3 entails an in-depth case study evaluation of three of the participating PET wards (one from each NHS Trust site) using semi-structured interviews with patients, carers and staff. Module 4 describes the development of a model and fidelity scale for PET using the information derived from the other modules with a working group of patients, carers and staff.

**Discussion:**

This is a feasibility study to test the application of the measures and methods in inpatient wards for older adults and develop a draft model for the intervention. The next stage will prospectively involve testing of the model and fidelity scale in randomised conditions to provide evidence for the effectiveness of PET as an intervention.

**Trial registration:**

ISRCTN31919196

## Background

The UK government has identified improving health and social care services for people with dementia as a national priority in a 5-year plan [1]. Over 700,000 people in the UK suffer from dementia, and this is projected to rise by 40 % by 2025 [2]. Two thirds of all UK hospital admissions of people over 65 and one quarter of all inpatients have dementia. As the level of cognitive impairment caused by dementia increases, so the way in which the person affected by dementia expresses their needs may change, with confusion and agitation as well as possible verbal and physical acts of aggression often presenting a challenge for carers. One factor frequently cited as contributing to agitation and other signs of distress in people affected by dementia, particularly within institutional care, is a lack of activity [3]. Behavioural and psychological difficulties are common in dementia and include confusion, agitation and possible aggression. Edvardsson and Nordvall [4] reported that people with dementia described boredom as part of their experience on a hospital ward. Pulsford [5] suggests that there is an absence of activities on wards for people with dementia as nurses may lack both time and confidence in their abilities to deliver them.

Protected engagement time (PET) is a relative newcomer among inpatient ward models intended to improve care delivery. It was first noted in mental health settings around 2004 [6] potentially arising from concerns in publications which reported that patients were bored, had little contact with staff and felt unsafe on acute wards [7, 8]. The Refocusing Model [9] contributed to the development of PET as an intervention through the subsequent decade and its use with older people on inpatient mental health wards has developed from this work.

The Refocusing Model had been implemented from 1999 in acute psychiatric wards in three National Health Service (NHS) trusts in England with reported benefits in terms of reduced staff sickness and absence, complaints, length of stay and use of formal observation. It involved increasing time for nurses to spend in one to one interaction with patients, an emphasis on nurses’ involvement in decision-making in their daily activities, regular use of clinical supervision and promotion of a calm environment. PET used some of these elements and placed the interpersonal relationship between staff and patients at the centre of ward practice by re-organising ward routines to increase staff and patient contact with minimal interruption.

Additionally, occupational therapists (OTs) have traditionally been seen as providers of activities for patients in mental health wards whilst the main staff group involved in PET has been nurses. This intervention may therefore provide an opportunity for task sharing between the professional groups and challenge existing professional boundaries. The intervention seems to have emerged from ward practice rather than from a well-defined theory or body of evidence and it has not been clearly defined and manualised. However, the Acute Care Collaborative report [10] provides the only source of guidance on implementing PET and identifies that

Regular times for PET are established: at least once a week and up to every day, for between one hour and half a day (between 3–4 hours in the morning or afternoon, not including night time).

During PET the ward is closed to visitors and professionals from outside the ward. Nurses are involved in the implementation of PET; whilst other ward staff participation appears to vary.

During PET ward staff do not make phone calls or administrative duties

Engagement may involve one to one meetings, group work, games or activities, or meals eaten together.

The same report describes positive feedback from the managers of services using PET, suggesting that it helps to create a calmer ward atmosphere. PET for older adult inpatients is a potentially attractive initiative as it may provide a calm yet stimulating environment and underlying distress such as agitation and other needs of patients affected by dementia, and other diagnoses, can be met effectively. However, robust evidence as to whether the difficulties in wards for older adults can be resolved by this means is still lacking, and there are some potential difficulties arising from PET, for example, the temporary exclusion of visitors to the ward may exacerbate the attachment needs of patients, depending on how ward staff choose to implement PET.

This study should have the potential to provide preliminary evidence as to the current level of use of PET, its effectiveness and clarification of its key components. The majority of data collection for the study will be carried out in three NHS Trust sites in England and will include all professional groups providing clinical care for patients. The intention of PET is to improve the amount of high-quality contact between ward staff and patients, with a potential benefit of increasing safety on wards through reducing incidents of falls and aggression. A description of nurses in the UK context includes both registered nurses and health care assistants (HCAs). The term ‘ward staff’ refers to nurses predominantly but also to medical staff, psychologists and occupational therapists that may be ward-based to varying degrees.

PET may also reduce distress and agitation without the use of psychotropic medication [11] which can have a significant effect on morbidity and mortality in older people [12]. The UK NHS spends £128 million annually on prescriptions for tranquiliser, hypnotic and antidepressant medications for people with dementia compared to £62 million spent on the two most commonly prescribed medications (tamoxifen and anastrozole) for the treatment of cancer across all health care settings [13]. PET could potentially reduce the use of tranquilisers and contribute to optimal utilisation of existing human resources, thus reducing NHS costs.

If the results from this study are positive then the uptake of PET may be increased, in anticipation of a larger trial of PET. If PET does not emerge as having positive effects this will also be important, as it will indicate that other paths to non-pharmaceutical solutions must continue to be sought.

### Study aims

This study aims ascertain prevalence of PET in older adult wards in England and to identify specific characteristics which contribute to the intervention. It also aims to assess the feasibility of the evaluation measures and methods used in the clinical setting to inform a larger randomised trial of the intervention. As PET has already been widely adopted, there is also a need for preliminary evidence as to how it has been implemented and whether there is evidence of positive benefits. We will compare a sample of wards where this model is currently used with otherwise similar local wards where it is not. The main research questions are:

1. How widespread is the use of PET in England on older adult mental health wards?
2. Do patients on older adult wards with PET spend more time in contact with staff than on other similar wards without PET?
3. Are there differences between patient experiences on wards with and without PET?
4. Are there differences between staff experiences on wards with and without PET?
5. Are there differences between rates of adverse incidents on wards with and without PET?
6. Are there differences between prescribing of tranquilisers on wards with and without PET?
7. How do patients, carers and ward staff experience PET, and what suggestions do they have about how to implement it?
8. What are the main components of PET?

Our primary hypothesis is that levels of agitation will be decreased and quality of life will be improved for patients on wards where PET is in place.

## Methods/design

The study uses a mixture of quantitative and qualitative methods and has four distinct, but overlapping, stages or modules.

1. Module 1: a service utilisation survey to determine prevalence of PET in older adult wards throughout England
2. Module 2: a prospective cohort comparison of patient and staff outcomes for a sample of five wards with and five without PET
3. Module 3: a qualitative exploration of the views of patients, carers and staff in three wards with PET
4. Module 4: development of a prospective model for the intervention.

A survey method has been selected to gather data on prevalence nationally as this is easiest to conduct within the study resources. We will use telephone interviews as they may yield a better response rate than on-line or postal returns and will allow the researchers to clarify any queries as they arise in the course of the interview [14]. The prospective cohort methodology used to compare outcomes for staff and patients in a smaller sample has been selected as it allows evaluation of routine current practice in two similar groups of wards. It benefits from being quick to implement and not requiring changes in practice or ward routine but has limitations in terms of potentially large confounding factors that are not controlled by randomisation [15]. Qualitative methods using individual semi-structured interviews provide the basis for the case study investigation of PET in three wards. The study uses this mixed methods approach as it appears most effective in evaluating complex interventions in health, such as PET [16, 17]. In developing the model for PET, we will combine frequency analysis from the service utilisation data with a concept mapping approach, which involves ranking components of the intervention in order of importance and relevance [18, 19].

The content of each module and their role in addressing the research questions are described in detail below.

1. Module 1: Service utilisation survey to determine prevalence of PET in older adult wards throughout England

This module will address our first research question above on the prevalence of PET in older adult wards in England, which will be ascertained through telephone interviews with the ward managers or their nominated deputies. The interviews will use a questionnaire largely based on that used in the PET acute study, with modifications to relate more specifically to the older adult setting. Research question 8 on the components of PET will be addressed in this module through collection of data on whether PET is implemented and in what way, in addition to information on ward staffing, organisation and activities. The frequency and duration of PET will also be established. The study is supported by the Clinical Research Network (CRN) which is a UK public-funded body assisting in the delivery of large scale competitively funded research studies [20]. Input from CRN researchers will facilitate identification of wards and initial contact with managers to provide them with basic information about the study and invite them to participate in the survey. Their responses to the telephone questionnaire will be entered directly into an online survey system, Qualtrics [21], by the researcher. Responses from the survey will be summarised using descriptive statistics.

2. Module 2: A prospective cohort comparison of patient and staff outcomes for a sample of five wards with and five without PET

We will employ a prospective cohort method to replicate that used in the preceding PET acute study, as this provides the most valid means of obtaining evidence for effectiveness of an already widely used intervention. This comparison comprises five sections: three sections will investigate the views and experiences of patients, carers and staff (research questions 3 and 4), the fourth will involve observational data on activities and interactions between staff and patients (research question 2), and the fifth will examine medication prescribing, incident rates (research questions 5 and 6) and staff sickness and turnover (research question 4). We will include 10 wards with approximately 15 patients per ward, and a low rate of turnover, giving an anticipated minimum number of 150 patients in total over the data collection periods.

### Comparison of the views and experiences of patients on wards with PET and those on wards without

#### Patient sample

All patients who have been in hospital for 14 days or more will be eligible to be included in the study, as the authors agreed that this time period would be adequate in which to have experienced life on the ward. There are two main types of in-patient service provision for older adults in England: wards specifically for those affected by dementia and other wards for all, irrespective of diagnosis. Both are represented in the study. We are unable to include patients who cannot communicate in English as the cost of translating documentation and hiring of interpreters is prohibitive within the study resources.

**Fig. 1:**
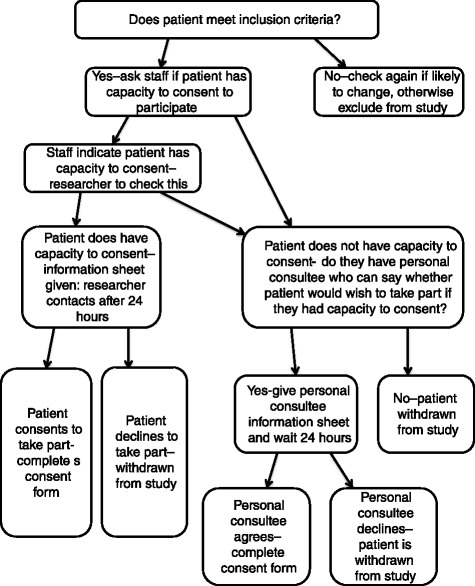
Capacity and consent flow chart for patients

Capacity to consent to participating in the study is checked by the researchers with clinicians involved in the patient’s care and with access patient notes. If a patient is thought to have capacity, the researchers (all of whom are experienced registered clinicians) will check during contact with the patient themselves [22]. If they lack capacity a consultee (a family member or friend who knows them well and has visited at least twice during their hospital stay) can be asked to participate on behalf of the patient [23]. Patients with capacity and consultees of patients without capacity will be given a copy of the information sheet. Up to 1 week, but ideally no less than 24 h, later, the researcher will contact the patient or consultee to answer any queries they may have about the study and ask if they are happy to proceed (Fig. 1).

For the purpose of calculating an appropriate sample size, Dementia Health-Related Quality Of Life (DEMQoL) [24] was identified as the primary outcome measure for this study. The effect size from a previous study using this measure [25] was 0.53 and 0.43 (mean 0.48). If we were to observe a similar effect, we would require 70 participants per group. However, this would ignore the clustering (by ward). If the sample size is inflated for this assuming an intra-class correlation (ICC) of 0.01 and 24 individuals per ward then this increases to 86 per group. If the ICC is 0.05, then it is increased to 149 participants per group but we do not have any information about this parameter. However, as this is a feasibility study, one of the aims is to estimate the parameters required for a formal sample size calculation for a full trial.

We aim to include a minimum of three assessment wards (one at each site) with the remainder treatment/long stay wards. We will include a total of 10 wards: five with PET and five without. We estimated the average length of stay from this combined group as 3 months, based on recent figures from one of the participating sites. We anticipate recruiting 30 patients from the longer stay wards, and potentially 135 from the three assessment wards, over 9 months. This would give us 165 in one group, or a total of 330, which would give adequate power for the DEMQoL.

Completion of the questionnaires for this module will be with assistance from the researcher. Demographic information on age, gender and ethnicity will be collated, as well as whether the patient is detained under the mental health act. The Client Satisfaction Questionnaire (CSQ) [26] was selected for ease of comprehension and effectiveness, as was the EuroQoL measure (EQ5D) [27, 28]. This measure can have three or five levels in response to each question or dimension. We have chosen the latter, EQ5D-3L, for brevity. The Camden Content of Care Questionnaire (CCCQ) [29] enquires about areas of help received during the hospital stay. Additional questions have been added to the CCCQ to ascertain whether the patient felt they required each area of help and, if so, whether they received it.

Nurses will be asked to complete the Cohen Mansfield Agitation Inventory (CMAI) [3] for each patient participant. The researcher will check that the completing nurses know the patients well and have had the opportunity to observe their behaviour over a few days to enable accurate ratings. They will also complete a DEMQoL for staff and an EQ5D-3L proxy version to enable their perceptions of the patients’ quality of life to be compared to those of the patient or their carer.

Researchers will complete three symptom rating measures with assistance from staff who know the patient. These are the Mini Mental State Examination (MMSE) [30], selected due to established effectiveness in measuring cognitive impairment in this population, the Functional Assessment Staging (FAST) [31] to measure stages of deteriorating functioning, and the Clinical Global Impressions (CGI) [32] to compare present and past levels of functioning. The tools have been selected for brevity and ease of completion, to minimise time required from clinical staff (Table 1).

**Table 1:** Patient related measures for module 2

Measure	Function	Completed by
Dementia quality of life (DEMQoL; patient or proxy version)	29 item measurement across 3 sub-scales of feelings, memory and behaviour, with a total score from 28 (good) to 112 (poor)	Patient or consultee
Client Satisfaction Questionnaire (CSQ)	8 item measurement of satisfaction with services and total score from 8 (worst) and 32 (best)	Patient
European quality of life—5 dimension (EQ-5D/EQ-5D proxy)	Measures perceived functioning in 5 areas—usual activities, mobility, anxiety/depression, pain/discomfort and self-care on three point scale for each	Patient or consultee
Camden content of care questionnaire (CCCQ)	Modified version of 21 item measure of help received whilst in hospital. Score from 0 (no help) to 147 (frequent)	Patient
DEMQoL (staff version)		Staff
Cohen Mansfield Agitation Inventory (CMAI)	Measures frequency on a scale of 1 (low) to 7 (high) of 29 behaviours with 4 sub-scales	Staff
Mini Mental State Examination (MMSE)	11 items testing five areas of cognitive function: orientation, registration, attention and calculation, recall, and language. Maximum score 30, with a score of 23 or lower indicative of cognitive impairment	Researcher
Functional Assessment Staging	Functional scale in 7 stages, from mild to severe incapacitation	Researcher
Clinical Global Impressions (CGI)	2 items a) Global severity, 7-point spectrum b) Global change, 9-point spectrum from improvement to deterioration	Researcher
Medication prescribing	Developed for the study to assess dosage and duration of prescribing for each medication	Researcher

#### Staff-related measures

We anticipate that within 10 wards there will be approximately 130 staff who can participate in this component of the study, based on an estimation of 20 staff per ward (including all professions), and a feasible response rate of 65 per cent based on a previous study of staff in mental health inpatient wards [33]. Consent will be implied by completion of the questionnaire.

The questionnaire will be distributed by the researcher to all staff working on the ward and will gather data on age, gender, ethnicity, profession and years in post. Levels of personal accomplishment, emotional exhaustion and depersonalization will be evaluated in the three sub-scales of the Maslach Burnout Inventory (MBI) [34]. Staff perceptions of the ward environment will be assessed through the Ward Atmosphere Scale (WAS) [35]. A measure developed by the authors for a previous investigation of PET in acute wards will be included to identify experiences of negative events at work and the impact of these on the staff member. Other questions relate to the degree of autonomy staff feel they have in their role and the support available from their colleagues. Scores for each of these questions will be examined individually (Table 2).

**Table 2:** Staff related measures for module 2

Maslach Burnout Inventory (MBI)	22 items with 3 sub categories. Scoring per category rather than total
Ward Atmosphere Scale (WAS)	Brief version of 40 items in 10 sub-categories, 4 items in each
Negative Events Scale	7 items asking whether adverse events were experienced and their impact on the staff member
Autonomy and support questions	22 items developed for the study to measure levels of staff autonomy and support from colleagues

#### Carer-related measures

Carers of patients that have been on the ward for a minimum of 14 days and have visited them at least twice on the ward will be eligible for participation in the study. They will be identified by staff and, provided that the patient does not object to the carer being contacted, will be approached by the researcher to participate either in person or by telephone. Informed written consent will be obtained. We anticipate including 100 carers in the study. Their age, gender and ethnicity will be recorded, and they will be asked about the burden of having a relative or friend who is unwell using the Caregiver Burden Scale [36]. In addition, they will be asked some questions developed for the study relating specifically to ward activity, organisation and their satisfaction with their relative’s treatment (Table 3).

**Table 3:** Carer related measures for module 2

Caregiver burden	21 items with 3 sub categories each with 7 items
Questions developed for the study	Measuring how staff spend time with patients, whether this is adequate and whether they feel safe on the ward

### A comparison of contact and interactions between staff and patients

Contact between staff and patients will be assessed using two measures: the Camden Staff-Patient Activity Record (CaSPAR) [37] and The Interaction-Observation Checklist (IOC) [38].

The CaSPAR was developed for a large-scale study of alternatives to acute inpatient care in England [36] and uses observation to measure the proportion of patients in contact with staff at pre-defined recording times. This will provide a comparison of the mean proportion of patients in contact with staff at PET and non-PET services. There are 28 recording times in total, with a recommended maximum of 2 per day and 10 per week. Our hypothesis is that the mean proportion of patients in contact with staff will be higher on PET wards than on others, and this will not be confined to specific times when PET is operating on the ward.

The IOC is an observational measure recording the activity of staff at 5-min intervals during pre-defined recording periods. Each staff member is recorded as undertaking one of five potential activities: interacting with patients, interacting with staff, engaged in solitary, task orientated or other activities. Interactions with patients are rated as positive, negative or neutral. This will provide a comparison of staff activity and number and quality of interactions with patients on PET and non-PET wards. The recording periods will span a 12-h period of ward activity over several days, with 1 h recorded each time. This time span will ensure that periods with PET and without PET are included on PET wards.

Both the CaSPAR and the IOC will be rated by the researcher over 3 to 4-week periods on each ward. In order to inform patients about observations of interactions on the ward the researchers will verbally explain what they are planning to do either at ward groups or individually. Additionally patients, staff and carers will be informed of the planned research through placing information posters in highly visible areas of the ward. The posters will contain a photo of the researcher(s), information on the study, on what the researcher will be observing on the ward, which will be head count (CaSPAR) and whether staff and patients are interacting (IOC). Patients and/or carers who object to being observed will be asked to inform the clinical team or the researcher of this, and their data will then be taken out of the study. Information leaflets detailing similar information to that in the posters will be available to carers via staff on the ward. These provide a further means of ensuring that carers are informed about the study and have the opportunity to request that their relative is not observed during this component.

### Service level data

Rates of adverse incidents on the wards will be evaluated using routinely recorded data for falls, aggression and self-harm, among others. One of the aims of PET identified by the Acute Care Collaboration was to facilitate safer ward environments, which can be measured by rate of adverse incidents. Staff sickness and absence and the median length of stay for patients will also be recorded as potential indicators of staff wellbeing and treatment effectiveness.

As PET may have an impact on the ward environment, and therefore on patients’ mental state, it may also indirectly affect the use of prescribed medication. We will therefore record the use of minor and major tranquilisers not only for patients who consent to being in the study but also for all patients on the wards for 1 week during the data collection phase of module 2. Obtaining consent from all patients for this component of the study would not be feasible, yet we believe it would be valuable, in the same way as the data from observations recorded with the CaSPAR and IOC. We consulted a variety of sources regarding ethical procedures in situations where obtaining individual consent is not feasible. In particular, we found valuable guidance on this in the Medical Research Council’s ethical guidance on ‘Personal Information in Medical Research’ [39]. This suggests that research use of information about patients without their informed consent may be valid where obtaining such consent is not feasible, where the study has no effect on care received by the patients and does not in any way directly involve them, and where an ethics committee has given its approval to such use of information. In this component of the study, no direct participation is required from patients, nor is any data collected about individually identifiable patients.

Written consent from patients or staff present will not be collected on the ward when observations are carried out. We will include all patients and staff in this component of the study and have been guided in this also through the Medical Research Council (MRC) ethical guidance. Our planned approach replicates that used in the PET acute study, during which we found that no staff or patients on any of 24 wards where the study was conducted objected to the process. However, we are aware that some staff and patients may have felt pressure to participate. Whilst trying to reduce the potential for this element of the study to be experienced as coercive, we cannot eliminate the possibility that it might be felt as such.

### Data collection, analysis and storage

Quantitative patient report measures will be completed on paper case report forms (CRFs), with a researcher, as a structured interview. Staff report measures will be distributed to staff by researchers, and they will be asked to return them to researchers in person or using a stamped addressed envelope. The experience of the English national staff morale study [33] was that allowing several weeks in which staff could return the questionnaires contributed to good response rates, as the researchers could prompt and encourage returns within this period. We will therefore ask staff to return the questionnaires during the data collection period on each ward, where the researcher will be available to prompt and assist if needed.

Staff completed measures on patients will be distributed to staff by researchers in person and collected in person when possible, within 1 week, which will allow the researcher and staff member to go through the measures and address any issues that may have arisen. The carer questionnaires will be completed with the researcher either over the phone or in person on the ward.

All quantitative data will be entered in anonymised from by researchers into an electronic database in a password-protected file using SPSS software, version 22. SPSS data will be converted into STATA software for analyses where adjustment for clustering is required. Linear regression analyses will be used to explore whether implementation of PET appears associated with outcomes, adjusting for clustering by service and (for staff and patient outcomes) known characteristics of respondents that are potential confounders.

The proxy version of the EQ5D will be used with a family carer (if available) and a nurse in order to assess the change seen from both perspectives. This is important as previous research into the proxy use of the EQ5D in dementia has shown that construct validity is better among clinical staff for observable dimensions, such as mobility and self-care, whilst family members had better construct validity on the harder to observe dimensions such as anxiety/depression [40]. The authors suggest that a more valid health state description might be achieved by using well-matched assessments from different respondent perspectives. The usefulness of the EQ5D compared to other study measures will be evaluated, for example, whether we can detect differences ratings between types of ward or different patient characteristics. We will also evaluate the ability of the patient and proxy EQ5D to detect differences between PET and non-PET wards.

### Cost-effectiveness component

The current study contains a health economic component which is intended to both inform any future study and to explore the potential cost-effectiveness of PET. A detailed ward-based audit will record duration and frequency of PET activity in addition to numbers and type of ward staff present during PET. In addition, data collected on adverse events, use of medicines and length of stay will be costed using appropriate unit cost data. Together with data on EQ5D, this will allow us to explore scenarios looking at potential costs and consequences of PET and to explore the potential for PET to be cost-effective.

3. Module 3: A qualitative exploration of the views of patients, carers and staff in three wards with PET.

This module will address research question 7 in collating the views of three stakeholder groups in relation to their experience of PET. It will also contribute research question 8 in identifying the components of the intervention. Thirty six in-depth case study evaluations will be conducted with four patients, four carers and four staff members from three of the participating five PET wards (one ward from each site). Non-PET wards will not participate in this module, which will overlap with the quantitative evaluation data collection time period in module 2. Staff and carer participants will be purposively selected so as to access the widest diversity and range of conceptually relevant characteristics of the types of staff and patient groups in each ward in terms of profession, gender, ethnicity, type and level of diagnosis and health status. Carers will therefore be selected not on the basis of their own characteristics but on those of their relative. The interviews for all groups will provide discursive and contextual information in the form of stories of implementation, process and outcomes. These will help identify which aspects of quantitative measures employed may be most relevant to participants’ experiences of PET as well as highlighting additional areas of impact not covered in the quantitative component and contextualised within the different PET wards studied.

- Patients

Patients will provide a vital narrative on the lived reality of PET, what happens on a practical basis, the impact of PET on them and on the ward and on their overall experience of hospital [41]. Topics to be explored will include their relationships with staff, how they spend their time on the ward, their relationships with other patients, and any effects that PET may have on their contact with visitors and with staff from outside services who work with them. (Appendix 1: semi-structured interview schedule for patients.)

### Inclusion criteria: patients

Patients consenting to take part in module 2 of the study, assuming their capacity has not changed, may be asked to participate in module 3 also. If a patient is deemed to lack capacity they will not be invited to participate in module 3.

b) Carers of patients

These may include relatives or friends of patients on the ward. We will use the term ‘carer’ to mean anyone, relative or friend, who is aware of the patients’ mental health difficulties and treatment, and has been involved in supporting them in some way. Their experiences on the ward in terms of access and contact with staff will be explored. Ward staff will be asked by the researcher to identify patients who have been visited by carers in the course of their admission (Appendix 2: semi-structured interview schedule for carers).

### Inclusion criteria: carers

We will only approach carers who have visited the ward at least twice, as we believe that less than this number might not allow an informed opinion of the service. Carers of all patients will be eligible to be included, not only those who have participated in either module 2 or 3.

c) Ward staff

Interviews will explore the impact of PET on the daily work and practice of staff, leadership of PET, their perceptions of benefits, and organisational issues that help or hinder PET and any ideas for changes to implementation.

### Inclusion criteria: staff

Only staff who have consented to take part in module 2 of the study by completing a questionnaire will be approached to take part in module 3. The sample will be selected purposively and will include at least one OT on each ward (Appendix 3: semi-structured interview schedule for staff).

The semi-structured interviews will provide an opportunity for themes to be introduced and developed by each participant, in telling their story in their own words of living, working on or visiting the ward before and whilst using PET. The interviews will aim to elicit rich detailed data and probe individuals specifically around issues related to changing patterns of activity, to gain understanding of why changes may occur, and identify key contributing processes and factors. Linkages and associations between factors affecting change will also be explored, giving a fuller picture to complement the quantitative data collected in module 2.

### Data collection, analysis and storage

All qualitative interview data will be audio recorded and transcribed in full. NVivo Version 11 software will be used to aid qualitative analysis. Concepts and categories from the interviews will be initially extracted using thematic framework analysis, which will allow participant-relevant issues to be explored and developed as themes on which to build an interpretation of the data [42, 43].

4.. Module 4: Development of a prospective model for PET.

This is an integral part of the study, addressing research question 8 on the components of PET and incorporating information from the module 1 survey and the views of researchers on the preceding acute study, as well as themes from the qualitative data from that study. The module will involve the developing of a working group of carers and people who use mental health services who will meet regularly to modify draft versions. The first meeting of the working group will take place before module 2 and an initial draft will be devised. The final version will have a comprehensive list of possible components of PET, and permutations for its use. The prospective model will therefore combine all possible elements of PET, in terms of frequency, duration, activities and staff involvement. Fidelity or adherence to this model will be measured through rating wards against the number of PET components present. The fidelity scale will present the strength of adherence to the model within each ward, which can be measured against patient and staff outcomes in a future trial of PET.

### Timescale

The study has been funded to be carried out over a 32-month period, from June 2013 to December 2015.

## Discussion

There is no information currently about where PET is implemented and in what way, nor what staff, patients and carers think of the intervention. It is one of several recently developed nurse-led interventions in mental health settings which focus on increasing time for engagement with patients in order to improve outcomes. The complexity of the ward environment and difficulties in accurately identifying contributory factors to patient and staff outcomes may have deterred interest in evaluating these interventions up to now. This study attempts to address this gap in evidence and determine the feasibility of replicating the methods and measures in a larger trial.

### Study strengths

Our programme and comprehensive mixed-methods design of research into PET will provide data from a wide variety of sources in order to develop a model and fidelity measure of PET. Detailed information from staff, carers and patients will be collected about their views of the concept through questionnaires and interviewing. The PET older adult study builds on the PET adult acute study through collecting data on tranquiliser prescribing on study wards and developing a model for the intervention. Collecting data on prescribing will give an indication of differences in prescribing activity between wards which do and do not provide PET. The national survey will provide evidence of what is currently being offered as PET and how prevalent uptake of PET is in these wards. These data are crucial to the development of the model.

We believe that the composition of the research team also adds to the study. Members of the team have already led an investigation of the impact of PET in adult acute wards, mentioned above, and this proposal arises from that work. FN is chief investigator on both studies, and the methodology and measures used are similar. RG also worked on both studies. Learning from the PET acute study has guided and potentially improved this work, and both can be seen as components in a programme to evaluate this intervention.

### Study limitations

As the PET older adult study is currently open, we can report on limitations and reflections on recruitment so far. Patient recruitment is proving more challenging in this study than in the sister PET acute study, in part due to the low numbers of patients with capacity to consent. Whilst we accounted for this in our study design through use of proxy measures, we have encountered some delays in obtaining consent from relatives due to infrequency of visiting, particularly in the sites outside London where there may be long distances between relatives’ homes and the hospital, thus limiting their ability to go to the ward. We also planned to include three assessment wards with an anticipated higher turnover of patients than longer stay wards. The assessment wards had closed in two sites by the time data collection commenced, and our access to new patients has been limited. In addition, the remaining assessment ward in one site has had a large cohort of patients who were discharged prior to the 14-day inclusion point, so this could not be included. We had not accounted for this in our planning, which was an important oversight.

## Conclusions

With PET emerging from ward practice rather than evidence and appearing to have become widespread over the past decade, our study of PET on older adult wards alongside the study already conducted on adult acute wards is important to gather the research evidence as to whether such a model improves quality of life and satisfaction on wards for patients, carers and staff. A key output from this work will be the development of a model for the PET intervention which can be tested in a later study. This will be of interest to clinicians and service planners as the results will be important in evaluating staff time, content of interactions with patients and costs to organisations.

## Appendix 1: Semi-structured interview schedule for patients

1. How have you found your time here on Ward X?
  - Prompt: Which aspects of ward life do you like?
  - Which aspects of ward do you dislike?
  **Questions about PET and other activities** (*The wording of the following questions will take into account how PET implemented and presented to patients*).
2. What do you think Protected Engagement time is/ what does the term mean to you?
3. Are you aware of Protected Engagement Time on this ward?
  - If no, describe PET and how it is implemented on the ward/what actually happens during PET
4. Does this time/activity seem any different from other times on the ward?
  - Prompt: In what ways?
5. How have you found the Patient Engagement Time activities/periods?
  - Prompt: What do you like about them/it?
  - What do you dislike about them/it?
6. What effect, if any, do you think Protected Engagement Time activities/period have on other aspects of your care or your stay on the ward?
  - *Prompt as many as relevant to how PET is implemented*:
  - Relationships with staff
  - Interactions with staff at other times
  - How you get on with other patients
  - General atmosphere on the ward
  - Access to professionals from other services
  - Visits from friends or family
  - Activities off the ward
7. Do you have any suggestions for how the PET activities/periods could be changed to make them better?
8. What other activities for patients are there on the ward?
9. Have you taken part in any these?
  - If no, can you tell me the reasons why?
  - If yes, how have you found these activities?
  **Questions about People on the Ward**
10. How do you get on with staff on the ward?
  - Prompt: primary nurse
  - Other nursing staff
  - Other members of the ward team
11. What is helpful about how staff interact with you on this ward?
12. What is not helpful about how the staff interact with you?
13. In what ways, if any, would you like the staff to be different?
14. How do you get on with other patients on the ward?
  **Ward Organisation and Atmosphere**
15. What do you think about how the ward is organised and run?
  - Prompt if necessary: e.g. mealtimes, medication rounds, ward rounds, visiting times etc.
16. Do you have any ideas for how this could be improved?
17. In general, how would you say it feels to stay on this ward?
18. Do you feel safe on the ward?
  - Prompt if necessary: What/who makes you feel safe/unsafe and why?
19. Would you like to add anything about any of the topics we’ve discussed, or anything else you think is relevant to Protected Engagement Time?

## Appendix 2: Semi-structured interview schedule for carers

1. Can you me tell approximately how often you have visited your friend/relative during their most recent admission to hospital?
  - Prompts: Time of day visits took place, day of week
  - For what purpose e.g. attending meetings with staff, bringing items in to relative, coming in for a chat,
2. What are your general impressions of the ward?
  - Prompt:
  - Friendliness of staff
  - Safety – of your relative, of others
  - Atmosphere- (e.g. relaxed, threatening, tense)
  - Noise levels
3. Would you change anything about the way the ward is organised?
  - Can you explain why?
4. How do you find visiting the ward? (How does it affect you?)
5. Have you heard of Protected Engagement Time (or whatever term is used in that particular ward)? (*If yes*) what do you understand by it?
6. Has PET affected you in any way during your relative’s admission?
  - If yes, can you say how?
7. What effect, if any, do you think PET has had to your relative’s care whilst on the ward?

## Appendix 3: Semi-structured interview schedule for staff

**Questions about PET and other activities** (*The wording of the following questions will take into account how PET implemented and presented to patients*)

1. What do you think Protected Engagement time is/ what does the term mean to you?
2. Are you aware of Protected Engagement Time on this ward?
  - If no, describe PET and how it is implemented on the ward/ what actually happens during PET
  - a. Does this time/activity seem any different from other times on the ward?
  - Prompt: In what ways?
3. How have you found the Patient Engagement Time activities/periods?
  - Prompt: What do you like about them/it?
  - What do you dislike about them/it?
  - a. What effect, if any, do you think Protected Engagement Time activities/period have on other aspects of patient care on the ward?
  - *Prompt as many as relevant to how PET is implemented:*
  - Relationships with staff
  - Interactions with staff at other times
  - General atmosphere on the ward
  - Activities off the ward
4. Do you have any suggestions for how the PET activities / periods could be changed to make them better?
5. What other activities for patients are there on the ward?
6. Have you taken part in any these?
  - If no, can you tell me the reasons why?
  - If yes, how have you found these activities?
  **Ward Organisation and Atmosphere**
7. What do you think about how the ward is organised and run?
  - Prompt if necessary: e.g. mealtimes, medication rounds, ward rounds, visiting times etc.
8. Do you have any ideas for how this could be improved?
9. In general, how would you say it feels to stay on this ward?
10. Do you feel safe on the ward?
  - Prompt if necessary: What/who makes you feel safe/unsafe and why?
11. Would you like to add anything about any of the topics we’ve discussed, or anything else you think is relevant to Protected Engagement Time?

## Abbreviations

CaSPAR: Camden Staff-Patient Activity Record
CCCQ: Camden Content of Care Questionnaire
CGI: Clinical Global Impressions Scale
CRF: case report form
CRN: Clinical Research Network
CSQ: Client Satisfaction Questionnaire
DEMQOL: Dementia Health-Related Quality Of Life Questionnaire
DENDRON: Dementia and Neurodegenerative Diseases Research Network
EQ5D: EuroQoL
FAST: Functional Assessment Staging
HCA: health care assistant
ICC: intra-class correlation
IOC: Interaction-Observation Checklist
MBI: Maslach Burnout Inventory
MMSE: Mini Mental State Examination
MRC: Medical Research Council
NHS: National Health Service
NIHR SDO: National Institute of Health Research Service and Delivery Organisation Programme
OT: occupational therapist
PET: protected engagement time
WAS: Ward Atmosphere Scale
